# Characteristics of the Electrophysiological Properties of Neuromuscular Motor Units and Its Adaptive Strategy Response in Lower Extremity Muscles for Seniors with Pre-Sarcopenia: A Preliminary Study

**DOI:** 10.3390/ijerph18063063

**Published:** 2021-03-16

**Authors:** Chia-Han Hu, Chia-Chi Yang, Shihfan Jack Tu, Ing-Jer Huang, Danaa Ganbat, Lan-Yuen Guo

**Affiliations:** 1 Department of Sports Medicine, College of Medicine, Kaohsiung Medical University, Kaohsiung 807, Taiwan; jiaalhan@gmail.com (C.-H.H.); jack.tu@ndorms.ox.ac.uk (S.J.T.); 2 The Master Program of Long-Term Care in Aging, College of Nursing, Kaohsiung Medical University, Kaohsiung 807, Taiwan; chiachiyang@kmu.edu.tw; 3 Center for Long-Term Care Research, Kaohsiung Medical University, Kaohsiung 807, Taiwan; 4 Oxford Orthopaedic Engineering Centre, Nuffield Department of Orthopaedics, Rheumatology and Musculoskeletal Sciences, University of Oxford, Oxford OX3 7LD, UK; 5 Department of Computer Science and Engineering, National Sun Yat-Sen University, Kaohsiung 804, Taiwan; ijhuang@cse.nsysu.edu.tw; 6 Biomechanical Research Laboratory, School of Mechanical Engineering and Transportation, Mongolian University of Science and Technology, Ulaanbaatar 14191, Mongolia; ganbatda@must.edu.mn; 7 Ph. D. Program in Biomedical Engineering, College of Medicine, Kaohsiung Medical University, Kaohsiung 807, Taiwan; 8 Department of Medical Research, Kaohsiung Medical University Hospital, Kaohsiung 807, Taiwan

**Keywords:** sarcopenia, motor unit, Decomposed Electromyography, aging

## Abstract

Older adults with sarcopenia, which is an aging-related phenomenon of muscle mass loss, usually suffer from decreases in both strength and functional performance. However, the causality between function loss and physiological changes is unclear. This study aimed to explore the motor unit characteristics of the neurological factors between normal subjects and those with sarcopenia. Five risk-sarcopenia (age: 66.20 ± 4.44), five healthy (age: 69.00 ± 2.35), and twelve young (age: 21.33 ± 1.15) participants were selected. Each participant performed knee extension exercises at a 50% level of maximal voluntary isometric contraction. Next, electromyogram (EMG) signals were collected, and information on each parameter—e.g., motor unit number, recruitment threshold, the slope of the mean firing rate to recruitment threshold, y-intercept, firing rate per unit force, and mean motor unit firing rate (MFR)—was extracted to analyze muscle fiber discrimination (MFD). Meanwhile, force variance was used to observe the stability between two muscle groups. The results suggested that there was no difference between the three groups for motor unit number, recruitment threshold, y-intercept, mean firing rate, and motor unit discrimination (*p* > 0.05). However, the slope of MFR and firing rate per unit force in the risk-sarcopenia group were significantly higher than in the young group (*p* < 0.05). Regarding muscle performance, the force variance in the non-sarcopenia group was significantly higher than the young group (*p* < 0.05), while the risk-sarcopenia group showed a higher trend than the young group. This study demonstrated some neuromuscular characters between sarcopenia and healthy elderly and young people when performing the same level of leg exercise tasks. This difference may provide some hints for discovering aging-related strength and function loss. Future studies should consider combining the in vivo measurement of muscle fiber type to clarify whether this EMG difference is related to the loss of muscle strength or mass before recruiting symptomatic elderly participants for further investigation.

## 1. Introduction

Over the past few decades, there has been a global demographic transformation, and people now live longer than before. These demographic changes have primarily been due to advances in public health and modern medicine that have reduced early-life mortality and the rate of infectious diseases. Virtually every global country is entering an aging society; people aged 60 or over are projected to increase from 962 million to more than 2 billion between 2017 and 2050 [1]. Numerous people are living longer with one or more chronic medical conditions and living with sarcopenia. The differential diagnosis of sarcopenia and frailty is a thoroughly onerous expression of aging. Sarcopenia is associated with adverse individual physical and metabolic changes contributing to morbidity and mortality [2]. These symptoms may, in turn, accompany osteoporosis and enhance the risk of falls and hip fractures [3]. The prevalence rate of sarcopenia increased with age but is influenced by the used evaluation algorithm and the studied ethnicity, gender, and region. A systematic review reported that the prevalence rate differed for community-dwelling older people aged ≥60 years (up to 20%); individuals in non-Asian countries were more likely to be sarcopenic than their counterparts in Asia [4], thus allowing for less uncertainty of the results of the disease screening.

Aging has been considered the primary cause of atrophy and muscle loss [5]. Skeletal muscle mass comprises approximately 40% of the total body mass and constitutes one of the body’s largest organ systems. Muscle atrophy can be overcome, at least in part, by physical rehabilitation and nutrition regulation [6,7,8,9]. However, these treatments do nothing to recover lost fibers; these age-related changes affecting skeletal muscles are likely to be related to declining numbers of motor units [10]. A motor unit is comprised of a motor neuron in the ventral cord, and the skeletal muscle fiber is innervated. There are approximately 60,000 motor neurons in the lower limbs [11], with each innervating hundreds or thousands of muscle fibers [12,13]. The available evidence indicates that if motor neurons are impaired or degraded during aging, the muscle may lose its innervation and be vulnerable to apoptosis [14]. Additionally, many studies have reported a substantial decrease in muscle fiber size in elderly subjects, and the reduction in muscle fiber size is fiber type-specific, with 10–40% smaller type II fibers observed in muscle tissue collected from the elderly compared to those young controls [15]. However, it remains unclear whether the loss of skeletal muscle in sarcopenia is similar to normal aging or not.

The surface electromyogram (sEMG) is a technique that is widely used to infer the characteristics of a motor unit (MU) population. In the early stage, motor unit numbers decline with increasing age in humans from direct counts of neuron cell bodies in the spinal cord’s anterior horn in autopsy samples [11,16]. Modern decomposition electromyogram (EMG) techniques reveal decreasing motor unit numbers of anterior tibialis with aging [17,18]. The neuromuscular system is highly adaptable via compensatory remodeling, as nearby motor neurons’ branching reinnervates recently denervated muscle fibers to preserve rudimentary needs [19,20]. An EMG can differentiate muscle types, such as the proportion of type 1 vs. type 2 muscle fiber pattern types of MU recruitment [21], but few studies have used sEMG decomposition techniques for the diagnosis of sarcopenia. Despite considerable interest, little is known about how motor unit remodeling is associated with sarcopenia.

Therefore, the present study aimed to compare motor unit-related parameters (e.g., motor unit number, recruitment threshold, and firing rate), muscle performance (force variance), and differentiated muscle type between young, healthy older, and sarcopenia men in the lower limb. This study sought to determine whether different motor unit types change with sarcopenia. This study tried to use exact and non-invasive measurements to observe sarcopenia, which is had by billions of human beings worldwide. We also applied the viewpoint that muscle composition would differentiate with aging, mainly via a decrease in type II muscle fiber and a relative preservation of type I muscle fiber. We endeavored to make our methodology be able to differentiate muscle fiber through EMG, which is more comprehensive and directly related to an early diagnostic screening. This information may provide a clinical basis for screening classification and preventing sarcopenia.

## 2. Materials and Methods

Twenty-two participants were recruited and included in the study. According to the Asian Work Group for Sarcopenia (AWGS), the diagnostic criteria are defined as a slower walking speed (<0.8 m/s) or weaker strength (grip < 26 kg for men, <18 kg for women) in combination with a low muscle mass (defined as appendicular muscle mass (ASM)/ht^2^ ≤ 7.0 kg/m^2^ for men and ≤5.4 kg/m^2^ for women) [22]. Individuals were divided into three groups: non-sarcopenia group (NS; 3 male and 2 female; mean age of 69.00 ± 2.35), risk-sarcopenia group (RS; 5 female; mean age of 66.20 ± 4.44), and young group (YG; 8 male and 4 female; mean age of 21.33 ± 1.15). Inclusion criteria were: aged 20–30 or 60–75 years (Table 1). Exclusion criteria included: individuals with a history of cancer (non-fatal cancers, e.g., skin cancer, stable prostate cancer, and other stable cancers with a good prognosis), hypercalcemia, or chronic renal failure in the last 5 years; uncontrolled high blood pressure (>150/90 mmHg); cognitive dysfunction; and musculoskeletal disorders in the six months before the investigation. The study procedures were approved by the Institutional Review Board of Kaohsiung Medical University Hospital with ethical approval number KMUHIRB-F(II)-20180058. All participants provided written consent before the study and then completed the Physical Activity of Senior Elder (PASE) survey and the International Physical Activity Questionnaire (IPAQ). The experiment terminated immediately if any incident occurred or discomfort was reported to ensure the safety of participants.

The AWGS developed a series of physical performance test approaches, including those for gait speed, hand-grip strength, and muscle mass, to discriminate sarcopenia. Participants were asked to perform a 6-m walking test three times; scores were averaged and recorded. Hand-grip strength (Jamar^®^ Hydraulic Hand Dynamometer-5030J1, Chicago, IL, USA.) was measured three times for each hand, and the maximal score was recorded [23]. We measured muscle mass by bioimpedance analysis (BIA) (Inbody230, Biospace Co., Seoul, Korea). Participants were required to obey the following: they had to (1) fast for over 3 h, (2) not ingest alcohol or caffeine for 12 h prior, (3) not exercise before testing, (4) not use lotion on the hands or feet, and (5) not were metal accessories. Muscle mass is represented by ASM (kg/m^2^):

$$\frac{ASM}{Ht2}=\;\frac{Appendicular\;skeletal\;muscle\;}{Squared\;height}$$

Total appendicular skeletal muscle is divided by squared height (*Ht2*).

To assess the knee extensor maximal voluntary contraction (MVC) force, the participant’s dominant leg was fastened with the hip and knees flexed at 90 degrees. The experimental set-up comprised a custom-made knee ergometer (Monitored Rehab System B.V., Netherlands). The body and leg were held in place with straps on a pad that was connected in series with a calibrated load cell (BAB-100M, Transcell Technology Inc., Nanjing, China). Before testing MVC, participants performed a standardized warm-up of several contractions, after which they were asked to perform a maximal effort lasting 3 s, with real-time visual feedback and verbal encouragement provided. This was repeated twice more with 3 min rest intervals between efforts; the highest of the three values was accepted as the MVC.

**Figure 1:**
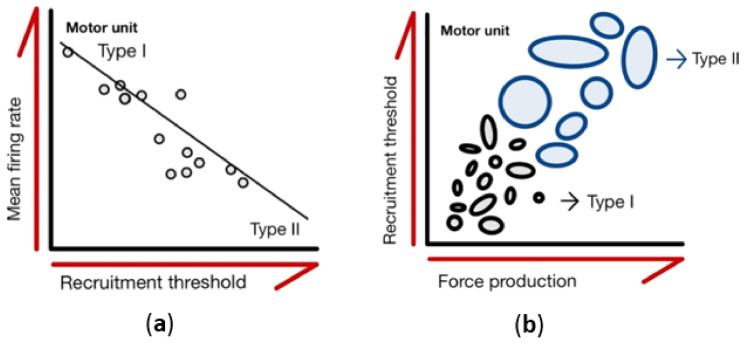
Characteristics of motor units. (**a**) Motor units are recruited from the smallest to largest with increasing force production. Each circle represents a motor unit, and the area of each circle shows the different sizes of the motor unit pool. (**b**) The mean value of the motor unit firing rates plotted as functions of recruitment threshold. Regression lines are drawn through the data from individual contractions, with each data point representing an individual motor unit.

Non-invasive multi-channel sEMG signals were detected through a single four-channel sensor (Bagnoli 16-channel EMG system, Delsys Inc., Boston, MA, USA) placed on the surface of the skin. Single sensor multi-channel sampling and associated algorithmic decomposition were used to measure the decomposed EMG (dEMG) [24,25]. The firing events of the MU were extracted from the EMG signals using the precision decomposition (PD) III algorithm [26]. The PD III was specifically designed for decomposing surface EMG signals into MU action potential trains [27]. The unique dEMG system allowed for assessments of MU firing properties and evaluations of different MUs within the recruited pool based on their recruitment threshold [28]. MUs that were extracted with a >90% accuracy were included in the final analysis [29]. For each MU, the mean firing rate (MFR) and recruitment threshold (RT) were calculated (Figure 1). The MFR was calculated in a 10-s interval in which the firing rate was stable, and the force was relatively constant at the steady-state. A 2-s Hanning window was applied to the MFR curves. The selected MU’s threshold force was calculated from the first firing event and then represented as the RT.

Subjects performed seated knee extension exercises at 50% of their MVC. During data collection, participants performed sub-maximal contractions following real-time output feedback displayed on a monitor. During the 50% MVC, sEMG signals were collected from the vastus medialis (VM) using a 5-pin electrode placed on the surface of the skin. The motor points of the VM were identified by a previous study [30]. The VM motor point was located about 4 cm above the patella and 3 cm from the medial condyle, over the muscle belly. The skin surface directly overlying the motor points was cleaned via shaving, removing superficial dead skin with adhesive tape, and sterilizing with an alcohol swab [31]. The dead layers of skin were removed with a body scrub applied to peel back multiple times to remove contaminants (Delsys, dEMG user guide). Torque data from the dynamometer (BAB-100M, Transcell Technology Inc.) were recorded by integrated hardware and software (EMGworks^®^ 4.0 Acquisition software, Delsys Inc., Boston, MA, USA)

**Figure 2:**
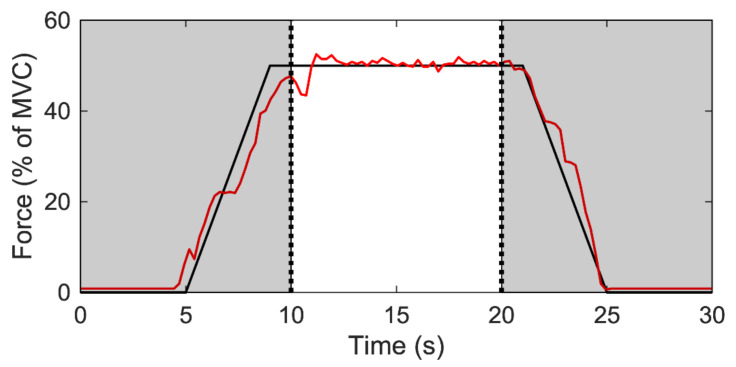
Steady contraction during 50% MVC. The black line represents the force output target; the red line represents actual measurement of subject’s muscle contraction force form the real-time monitor.

Sub-maximal contractions involved a 5-s quiescent period, a linear 4-s ramp-up in force from 0% to 50% of before-intervention peak MVC force, 10-s holding force levels constant at 50% of MVC force, a linear 4-s ramp-down from 50% to 0% of MVC force, and a final 5-s quiescent period. The total testing time was 30 s (Figure 2). For each participant and contraction level, linear regressions were performed for the MFR vs. RT relationships, with the y-intercepts and slopes (pulse per second; pps/%MVC) used for subsequent statistical analysis (Figure 1b). The y-intercept was considered to be the theoretical MFR for an MU recruited at 0% MVC, and the slope was considered to be the coefficient of MFR that decreased with increments in RT.

**Figure 3:**
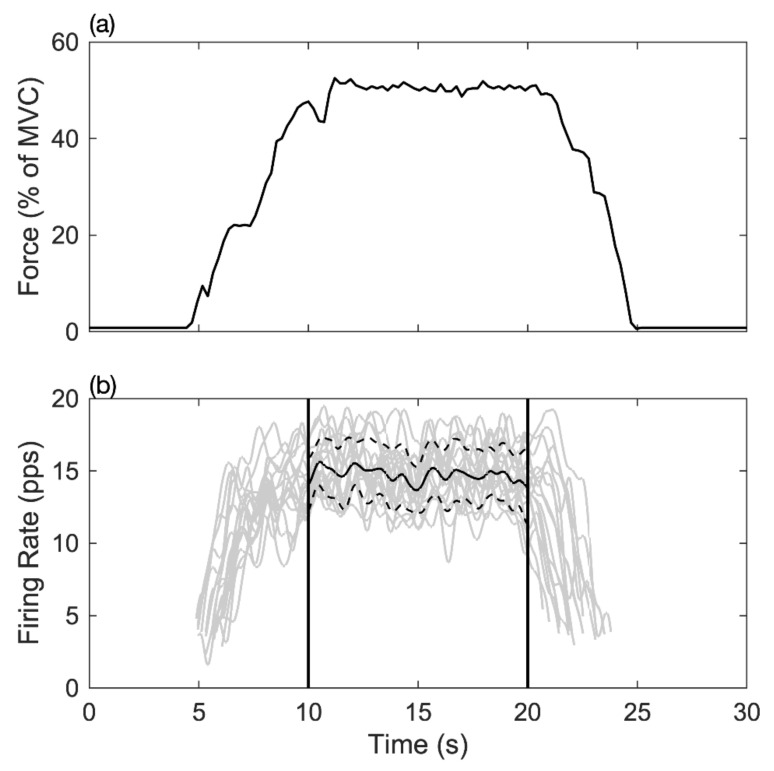
The relationship between muscle force output and motor unit MFR in the time-domain. (**a**) The line shows the muscle force output in the maximum voluntary contraction force percentage during the task. (**b**) Each grey curve represents the time registered firing rate of a motor unit during the contraction task; the short dash line indicated the range of one standard deviation, and the solid line represented the mean firing rate of all detected motor units between 10–20 s.

The MFR during the 10-s steady-state was further analyzed. Each participant’s MU firing rate was calculated separately. Each MU firing rate carried out time-domain integration. The specific MU with a time-domain integral below the MFR’s standard deviation was regarded as an MU that was independent of the steady contraction, which was excluded. The remaining MU is represented as the key for acting, named muscle fiber discrimination (MFD). The concept was based on a study by Potvin et al., who showed the prediction model of the motor unit firing rate during muscle contraction [32]. The time-domain integral of the MU firing rate of each MU is calculated using the equation:

(1) $$\int_{10}^{20}MUFR\cdot dt\;-\int_{10}^{20}LowerSTD_{MUFR}\cdot dt\;>\;0\;$$

(2) $$\int_{10}^{20}MUFR\cdot dt\;-\int_{10}^{20}LowerSTD_{MUFR}\cdot dt\;<\;0$$

where *MUFR* (motor unit firing rate) represents a single motor unit (Figure 3) and *LowerSTD_MUFR_* (lower than one standard deviation of *MUFR*) represents a standard deviation when performing a constant contraction. If an output meets Equation (1), we denoted it as a type I muscle fiber; otherwise, Equation (2) denoted it as a type II muscle fiber. In Equation (3), for the firing rate per unit force (pps/Newton) at a constant contraction period, is as follows:

(3) $$\mathrm{Firing}\;\mathrm{rate}\;\mathrm{per}\;\mathrm{unit}\;\mathrm{force}=\frac{Mean\;firing\;rate\;\left(pps\right)}{50\%MVC\;\left(N\right)}$$

To observe an individual’s muscle performance, measured as the force output stabilization during muscle contraction, the coefficient of variance of VM muscles at the steady-state contraction (Figure 2) was calculated using the equation:

(4) $$\mathrm{CV}=\frac{\sqrt{\frac{\sum {\left(x-\text{µ}\right)}^{2}}{n-1}}}{\frac{\sum_{t=10}^{20}x_{t}}{n}}$$

In Equation (4), x indicates the participant’s force output during the 10-s continuous period, µ represents mean force output, and n represents the number of participants.

Data are presented as mean (±SD). When data fit a normal distribution, between-group differences were compared using an ANOVA. When significant differences were observed, a Bonferroni post-hoc test was performed. When the data were not normally distributed, between-group differences were compared using a Kruskal–Wallis test. The SPSS Version 25.0 (IBM SPSS, Chicago, IL, USA) statistics software was performed for all statistical analyses, with statistical significance set at *p* < 0.05.

## 3. Results

Older participants were classified as non-sarcopenic and risk-sarcopenic. People found to have a crisis of sarcopenia were involved based on the AWGS. The characteristics of the subjects are summarized by age group in Table 1. Participants were 21.3–69.0 years of age. Body weight was significantly lower in the older age groups (*p* < 0.05). The mean strength (MVC) was the highest in the young group, lower in the older age group, and lowest in the risk-sarcopenia group (*p* < 0.05). Muscle mass was significantly lower in the older age groups (*p* < 0.05).

Table 2 presents no difference between the three groups for motor unit number, recruitment threshold, y-intercept, mean firing rate, and muscle fiber discrimination (*p* > 0.05). The slope and firing rate per unit force in the risk-sarcopenia group were significantly higher than in the young group (*p* < 0.05). Regarding muscle performance, the force variance in the non-sarcopenia group was significantly higher than in the young group (*p* < 0.05), and the risk-sarcopenia group had a higher trend than the young group. A significant firing rate per unit force in the VM was shown between groups (*p* < 0.05). The VM firing rate was significantly lower in the non-sarcopenia group than in the risk-sarcopenia group.

## 4. Discussion

This study examined the motor unit properties and force performance of people affected by sarcopenia. The results showed that the surviving motor units were compensated for by an increased mean firing rate to maintain the sarcopenic elderly people’s physiological needs. These findings suggested that the mean firing rate and force ratio may distinguish the elderly from early stage sarcopenia.

This study found a negative relationship between the motor unit recruitment threshold and the mean firing rate, indicating the more massive the recruited motor unit, the lower the mean firing rate it has, which is known as the ‘onion skin’ phenomenon [25,33,34]. A slope’s inclination is associated with muscle fatigue, thus indicating that the motor unit adapts to physiological responses. When the muscle is fatigued, the slope’s inclination and the y-intercept decrease [35]. For the muscle type, the larger the proportion of slow muscle fiber in the muscle, the smaller the slope the inclination produces. Our results indicated that the slope’s inclination in the old group was smaller than in the young group; sarcopenia may have severe degeneration.

One of the aims of this study was to observe the connection between force output and motor unit. Aging is accompanied by muscle strength decreases, with older adults producing more firing rates per unit force to maintain the same strength of target force output as young people [36]. Moreover, aging has been found to lead to a low recruitment threshold size and a firing rate increase [37]. In this study, the risk-sarcopenia group had the highest motor unit firing rate per unit force than other groups, and sarcopenia was found to be a precedent for the aging process. Additionally, old and young adults have generally been found to have similar type 1 fiber cross-section areas [38]. We suggested that the sarcopenia group had an increased firing rate per unit force due to muscle atrophy.

Neuron muscle fiber is initially benign when entering the aging process. The neuron is denervated, and then muscle fiber atrophies, with the motor unit number decreasing [14]. As mentioned above, these events may cause older adults’ skeletal muscle mass and muscle strength to decrease. The compensatory effect for increasing muscle force output may occur in two ways: (1) increasing motor unit firing frequency to augment force output or (2) increasing motor unit recruited numbers, with much more motor units required to complete the movement. In this study, the sarcopenia group’s mean firing rate was found to have a trend that was higher than the young group when performing at the same level as the target force. In other words, people who suffered from sarcopenia may require more motor units firing to carry out the same tasks as ordinary people. A previous study noted no difference between average young and older adults’ vastus medialis mean firing rate when performing unidentical isometric contraction level [39]; it was found that aging did not diminish the neuromuscular system during steady-state contraction, which may explain the mean firing rate change between groups in this study.

According to Henneman’s size principle, small motor units are first required, and as time continues or strength reinforces, larger motor units are involved in a task [40]. Additionally, there are two kinds of motor unit physiology: a smaller motor unit, which is slow-twitching, a type I muscle fiber, and fatigue-resisted, and a larger motor unit, which is fast-twitching and has type II muscle fiber fatigability [41]. In addition, during an isometric contraction, there is a negative correlation between the recruitment threshold and the mean firing rate; a higher RT has been found to respond to a lower MFR [28,42,43]. One study found that a type I motor unit’s MFR was higher than that of type II. Moreover, during isometric muscle contraction, motor neuron excitation is raised to counter muscle fatigue, and the force capacity declines at the same time [32]. In the present study, the motor unit discrimination of sarcopenia patients was higher than of healthy subjects, and aging was found to potentially accompany muscle fiber decay and atrophy, with fast muscle fiber degenerating to primarily preserve slow muscle fiber [44]. This study indicated a higher amount of type I motor units in sarcopenia patients than in healthy subjects, and the survival motor units were found to reinnervate the muscle fiber to maintain muscle size; however, many motor units were lost [45]. We observed that the sarcopenia and healthy groups’ muscle mass were significantly lower for the vastus medialis, indicating that people affected by sarcopenia might experience uncompleted compensatory remodeling and cannot preserve muscle fiber denervation, thus accelerating the decline of their muscle mass. Therefore, the compensatory remodeling of a type I motor unit might increase the mean firing rate to maintain activity physiologically. Though it is believed that specific exercise prescriptions or nutrition intake might alleviate muscle atrophy and mitigate their inevitable effects in the elderly [46,47], studies have rarely explored the issue by decomposing motor unit firing patterns in sarcopenia. Therefore, this study broke new ground and provides a guide for early diagnosis and exercise scanning as a starter.

There were several limitations to the present study. The first limitation was related to the study sample. The relatively small sample size limited our ability to detect subtle differences. Meanwhile, our statistical analysis results showed trends with no significantly differences, especially in clinical settings. A larger sample size would increase the diversity of age and gender, which may raise the confidence of this conclusion and the application for more diverse populations. The second limitation was that of the onset of fatigue in submaximal tests; it is believed that muscle fatigue may have subtly arisen in the present study, although all subjects were asked to rest until they felt able to continue the experiment. Thus, subjective rating scales (i.e., rated perceived exertion) and physiological monitoring may be tenable in future studies. The third limitation was related to age-associated changes in the motor unit. According to the Henneman size principle [40], larger, high-threshold motor units may not have been active and may have differed between groups. However, this had the added advantage that the active motor units would have mainly been composed of the earlier recruited type 1 muscle fibers.

On the other hand, aging is associated with neuromuscular junction (NMJ) remodeling, and aged NMJ expands with a larger motor endplate [48]. Furthermore, other research has proven that in aging, fast muscle fiber is retrograded and slow muscle fiber is relatively preserved [49]. In summary, these conditions that damage some motor units usually result in overall muscle weakness but high firing rates of individual motor units that are still intact.

To sum up, sarcopenia may be irrecoverable to type I muscle fiber, and most aging people would fall behind after getting this disorder; thus, we intended to reveal the trend of muscle atrophy in sarcopenia with MFD, which has the potential to form a greater understanding of the syndrome. This study’s scope was limited to observing the early stage of the elderly with sarcopenia. Moreover, a lack of evaluated muscle fatigue may have affected the trend. The effects of muscle fatigue, muscle composition changes (e.g., fiber type), and physical inactivity on age-associated changes in motor unit properties need to be assessed in future studies. We lacked subjects with severe symptoms for a cross-sectional study’s integrity. However, we provided a unique viewpoint for sarcopenia with a decomposed mean firing pattern.

## 5. Conclusions

It was shown that the firing rate per unit force for use in diagnosis screening may play some role in determining sarcopenia and preserving muscle activity. Overall, our results suggest that muscle fiber, which has a motor unit firing rate, may be a critical factor determining sarcopenia’s progression.

## Figures and Tables

**Table 1:** Anthropometry.

	RS Group (*n* = 5)	NS Group (*n* = 5)	YG (*n* = 12)
Age (yr)	66.20 ± 4.44	69.00 ± 2.35	21.33 ± 1.15 *^,a,b^
Gender	5 females	3 males, 2 females	9 males, 3 females
Height (m)	1.54 ± 0.03	1.59 ± 0.08	1.70 ± 0.10 *^,a,b^
Weight (kg)	50.00 ± 2.71	60.20 ± 13.85	65.59 ± 11.24 *^,a,b^
MVC (N)	161.26 ± 46.11	258.47 ± 69.25	491.93 ± 155.26 *^,a,b^
ASM(kg/m^2^)	5.28 ± 0.44	7.20 ± 1.40	7.81 ± 1.30 *^,a,b^
Gait speed (m/s)	0.83 ± 0.15	1.07 ± 0.11	-
Grip strength (kg)	20.00 ± 3.74	29.60 ± 8.88	-
PASE (score)	144.2± 3.44	183.40 ± 101.80	-
IPAQ (score)	-	-	315.17 ± 235.26

Abbreviation: NS: non-sarcopenia group; RS: risk-sarcopenia group; YG: young group; ASM, appendicular muscle mass; PASE: Physical Activity of Senior Elder; IPAQ: International Physical Activity Questionnaire; MVC: maximal voluntary contraction. *, represents *p* < 0.05; ^a^ = *p* < 0.05 difference from NS; ^b^ = *p* < 0.05 difference from RS.

**Table 2:** Motor unit parameters during steady-state for the vastus medialis.

	RS Group (*n* = 5)	NS Group (*n* = 5)	YG Group (*n* = 12)
Number of MUs (#)	23.40 ± 7.37	26.60 ± 6.80	20.33 ± 5.53
Slope (pps/%MVC)	−0.39 ± 0.06	−0.20 ± 0.25	−0.48 ± 0.17 *^,a^
Y-intercept (pps)	25.04 ± 2.65	21.02 ± 3.90	26.17 ± 5.62
Recruitment threshold (%)	23.32 ± 13.91	20.84 ± 12.42	22.97 ± 10.17
Mean firing rate (pps)	17.70 ± 3.18	15.92 ± 2.39	14.66 ± 1.83
Firing rate per unit force (pps/MVC (N))	0.24 ± 0.09 ^a^	0.14 ± 0.06	0.07 ± 0.03 *^,a,b^
Muscle fiber discrimination (pps)	19.16 ± 1.55	17.14 ± 3.20	15.89 ± 2.45
Force variance	5.4 ± 2.8	4.5 ± 0.8	1.4 ± 1.4 *^,a^

Abbreviation: NS: non-sarcopenia group; RS: risk-sarcopenia group; YG: young group; MU: motor unit; pps: pulse per second; MVC: maximal voluntary contraction; N: Newton. *, represents *p* < 0.05; ^a^ = *p* < 0.05 difference from NS; ^b^ = *p* < 0.05 difference from RS.
